# Multimorbidity in middle-aged women and COVID-19: binary data clustering for unsupervised binning of rare multimorbidity features and predictive modeling

**DOI:** 10.1186/s12874-024-02200-x

**Published:** 2024-04-24

**Authors:** Dayana Benny, Mario Giacobini, Giuseppe Costa, Roberto Gnavi, Fulvio Ricceri

**Affiliations:** 1 Centre for Biostatistics, Epidemiology, and Public Health, Department of Clinical and Biological Sciences, University of Turin, Orbassano, Turin, 10043 Piedmont Italy; 2 Modeling and Data Science, Department of Mathematics, University of Turin, Via Carlo Alberto 10, Turin, 10123 Piedmont Italy; 3 Data Analysis and Modeling Unit, Department of Veterinary Sciences, University of Turin, Turin, Italy; 4 Unit of Epidemiology, Regional Health Service, Local Health Unit Torino 3, Grugliasco, Turin, Italy

**Keywords:** Clustering, Sparse binary data, Binary classification, Multimorbidity, Machine learning, COVID-19, Public health, Predictive modeling, Long COVID, Unsupervised binning

## Abstract

**Background:**

Multimorbidity is typically associated with deficient health-related quality of life in mid-life, and the likelihood of developing multimorbidity in women is elevated. We address the issue of data sparsity in non-prevalent features by clustering the binary data of various rare medical conditions in a cohort of middle-aged women. This study aims to enhance understanding of how multimorbidity affects COVID-19 severity by clustering rare medical conditions and combining them with prevalent features for predictive modeling. The insights gained can guide the development of targeted interventions and improved management strategies for individuals with multiple health conditions.

**Methods:**

The study focuses on a cohort of 4477 female patients, (aged 45-60) in Piedmont, Italy, and utilizes their multimorbidity data prior to the COVID-19 pandemic from their medical history from 2015 to 2019. The COVID-19 severity is determined by the hospitalization status of the patients from February to May 2020. Each patient profile in the dataset is depicted as a binary vector, where each feature denotes the presence or absence of a specific multimorbidity condition. By clustering the sparse medical data, newly engineered features are generated as a bin of features, and they are combined with the prevalent features for COVID-19 severity predictive modeling.

**Results:**

From sparse data consisting of 174 input features, we have created a low-dimensional feature matrix of 17 features. Machine Learning algorithms are applied to the reduced sparsity-free data to predict the Covid-19 hospital admission outcome. The performance obtained for the corresponding models are as follows: Logistic Regression (accuracy 0.72, AUC 0.77, F1-score 0.69), Linear Discriminant Analysis (accuracy 0.7, AUC 0.77, F1-score 0.67), and Ada Boost (accuracy 0.7, AUC 0.77, F1-score 0.68).

**Conclusion:**

Mapping higher-dimensional data to a low-dimensional space can result in information loss, but reducing sparsity can be beneficial for Machine Learning modeling due to improved predictive ability. In this study, we addressed the issue of data sparsity in electronic health records and created a model that incorporates both prevalent and rare medical conditions, leading to more accurate and effective predictive modeling. The identification of complex associations between multimorbidity and the severity of COVID-19 highlights potential areas of focus for future research, including long COVID and intervention efforts.

## Background

Multimorbidity, which refers to the presence of multiple diseases and medical conditions in one individual, is consistently linked to a lower health-related quality of life in mid-life [1, 2]. Additionally, there is evidence suggesting that women have a higher likelihood of developing multimorbidity compared to their male counterparts [3]. Moreover, having multiple health problems at the same time has been found to make healthcare more expensive and create difficulties for healthcare systems in terms of resource allocation and providing appropriate care [4].

Moreover, multimorbidity can worsen the effects of long COVID in several ways [5, 6], when multimorbidity is present, additional symptoms related to other chronic conditions can compound the overall symptom burden, making it more challenging for individuals with long COVID to manage and recover from their illness. Research studies have indicated that individuals with multimorbidity have been adopting various precautionary behaviors during the pandemic [7, 8]. This is reflected in the restrictive guidelines recommended by authorities to control transmission [9]. Furthermore, studies have found that females are more likely to adopt protective measures compared to males [8]. The difference in precautionary behaviors based on gender underlines the importance of considering various demographic factors in the development of public health interventions during a pandemic.

This study specifically focuses on clustering binary data related to various medical conditions in middle-aged women. Cluster analysis is a valuable statistical technique for grouping objects based on their similarity in terms of indicator variables or features, and can be applied to identify clinically significant multimorbid groupings of medical conditions [10]. By using cluster analysis, researchers can learn important information about how different medical conditions are related and occur together. This helps them understand the complex connections between diseases and to develop personalized ways of treatment. It is also evident from the existing studies that clustering methodology can be applied to identify patient subgroups with similar disease profiles or symptom patterns [11]. Furthermore, it also can be utilized for identifying patient subgroups with distinct healthcare utilization trends and identifying risk factors associated with adverse outcomes [12]. In a multimorbidity study [13], the authors utilized K-means non-hierarchical cluster analysis to identify patterns of multimorbidity. Similarly, another study [14] focused on stratifying a population of high-risk multimorbid patients by using cluster analysis for risk stratification and identifying distinct characteristics of each cluster. These findings emphasize the significance of healthcare reform in addressing the unique needs of different patient clusters. By tailoring interventions and care strategies based on these identified clusters, healthcare providers can effectively address the diverse challenges associated with multimorbidity. Self-Organizing Feature Maps (SOFMs) have been widely employed in various clustering applications, including tasks like handwritten digit recognition [15]. In another study [16], the authors employed SOFMs to identify clusters of patients based on their healthcare data. However, SOFMs are not commonly used for clustering multimorbidity patterns, as these patterns typically involve clinical and demographic data rather than image data. Instead, other clustering approaches such as k-means, hierarchical clustering, and latent class analysis are more commonly employed for multimorbidity clustering.

However, in our study, we focus solely on clustering rare features, which are medical conditions that are not commonly observed in patient data. The methodology section explains the procedure employed in this study, detailing the process of grouping multimorbidity features into bins using a matrix based on cluster structures. This process involves two levels of clustering: the feature level and the data level, without making assumptions about the number of feature clusters. Once the features associated with each cluster are identified, they are mapped to corresponding bins. The unsupervised bins are then merged with prevalent features to create a new engineered feature matrix. The performance of models using clustered data is compared to models without clustered data, and the importance of the features is investigated, leading to the interpretation of the models.

## Methods

The study focuses on a cohort of females in Piedmont, Italy. The study examines their medical history to analyze their multiple health conditions prior to the Covid-19 infection. The multiple health conditions are derived from the data of prescribed medications and diagnosed diseases. Prescribed medications are considered multimorbidity features in this study. Moreover, polypharmacy often goes hand in hand with multimorbidity, as individuals with multiple chronic conditions may require a complex medication regimen to manage their health. The severity of Covid-19 in these patients is determined based on whether they were hospitalized due to Covid-19 or not. Each patient’s information in the dataset is represented as a binary vector, indicating the presence or absence of various health conditions. Since many health conditions are very rare and present in some patients only, the data is sparse. By grouping and analyzing this sparse medical data, new composite features are created from rare features and combined with the existing common multimorbidity features including age to develop a predictive model for Covid-19 severity.

### Study design and study population

This study is designed as a retrospective cohort study as it involves the retrospective analysis of data to examine the association between multimorbidity and COVID-19 outcomes over time. The historical data on prescriptions and hospital history from 2015 to 2019, which can be considered as exposure variables (multimorbidity) over a period of time. This study is evaluating the outcome of interest, which is the COVID-19 hospitalization status, during the period from February 2020 to May 2020.

Out of the 1,918,549 individuals in Piedmont, aged between 45 and 74 years, 85,348 underwent at least one swab during the observation period from February 2020 to May 2020 [17]. Of the 12,793 individuals who tested positive, 6832 females were there.

The study focused on a specific subset of the population, namely female patients aged 45-59 residing in Piedmont, Northern Italy. Inclusion criteria comprised individuals who tested positive for COVID-19 during the observation period from February 2020 to May 2020. Exclusion criteria included males, individuals outside the age range of 45-59, those residing outside Piedmont, and individuals who did not test positive for COVID-19 during the specified time frame. The corresponding exclusion and inclusion criteria resulted in 4,477 observations of female patients, and only 13.8% of them were hospitalized due to COVID-19 during the observation period.

### Dataset and features

The data used in this study were collected from the Piedmont Longitudinal Study (PLS), utilizing administrative databases that involve linking anonymous records at the individual level [17]. This study investigates the multimorbidity profile of 4,477 female patients aged 45 to 59 years. We classify individuals aged 45 to 59 years as belonging to the middle-age category, aligning with prior research in this context [17].

In the dataset. there are 195 input features and 1 outcome variable where data comprises of 4,477 patient records (4477,196) where 3,861 individuals are not hospitalized and 616 individuals are hospitalized due to COVID-19. Since this is unbalanced data, the data is randomly undersampled. The under sampled dataset comprised of 1,232 patients’ records (4477,196). After resampling, zero columns are eliminated, which corresponded to records where all variables had a value of 0 in the resampled dataset. Subsequently, a comparison is conducted on all remaining fields in the resampled data with the original data to assess the similarity of proportions.

It is crucial to examine whether the resampling has been correctly accomplished as it is an important step in training this data using Machine Learning. So, the exclusion of features with significant differences is performed for retaining only those features for which there is no statistical evidence of a significant difference in proportions between the original and resampled datasets. The statistical procedure used to compare the proportion of each feature in the resampled data with that in the original data is based on a one-proportion z-test. By performing this method on each feature individually and eliminating the features in the resampled data that exhibit a significant difference in proportion compared to the original data, a new resampled dataset is created (1232,175) that is statistically similar to the original data. The resulting statistically similar dataset comprised of 1,232 patients records with 175 variables including the outcome variable.

The features used in this study are the prescriptions and diseases diagnosed for each patient in the cohort, along with the age variable. The age variable is represented as a binary variable, where 1 represents patients aged over 53 and 0 represents patients aged 53 or under. Specifically, the threshold of 53 was chosen to reflect the median within the age range, providing a meaningful criterion for distinguishing between patients above and below this central value in our analysis. All features are represented as binary variables, where 1 indicates the presence of the condition and 0 indicates the absence of the condition in the patient’s medical history from 2015 to 2019, prior to the COVID-19 pandemic. The data used in this study is labeled and belongs to the binary class of non-hospitalized and hospitalized patients (0 and 1, respectively).

### Unsupervised feature binning

**Fig. 1:**
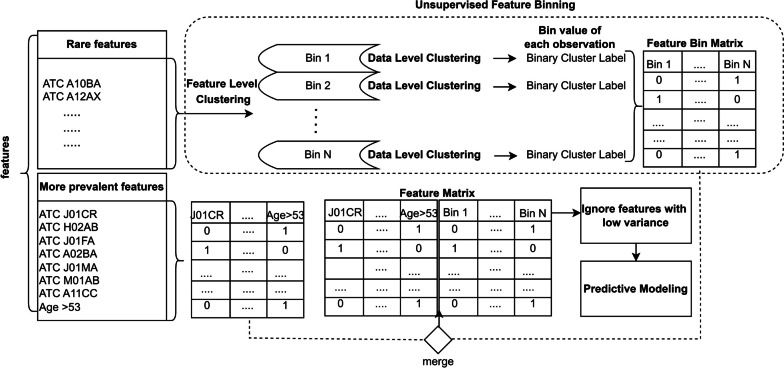
Feature level is performed to assign features into different clusters which are the Bins and data level clustering is performed where patients’ records are grouped into clusters based on the features within each Bin before predictive modeling

To group the multimorbidity features into various bins, a matrix is reconstructed based on the cluster structures. The clustering process involves two levels: feature level and data level, as shown in Fig. 1.

Binary Matrix Decomposition (BMD) offers a powerful approach for unsupervised feature reduction in binary data settings [18]. BMD seeks to factorize given binary data matrix into a reduced feature space that represent data points (U) and another reduced feature space that represent features (V). By projecting the original data points onto the reduced space determined by U, BMD achieves dimensionality reduction [19]. The latent factors in U capture underlying patterns or hidden structure in the data, providing a compressed representation suitable for various tasks like clustering. Thus, by decomposing the data into lower-dimensional binary matrices, BMD identifies latent features and reduces dimensionality while retaining essential information.

At the feature level clustering, a general model for clustering binary data that use the Binary Matrix Decomposition algorithm [20] is used to assign features into different clusters without bootstrapping on labeled train data. This method uses a Binary Matrix representation where rows represent the patients’s records, and columns represent features. BMD algorithm decompose the binary matrix to yield a probability matrix indicating the likelihood of features being part of specific clusters. Features are assigned to clusters iteratively based on whether the corresponding probability exceeds a threshold, refining the clustering model through repeated optimization until convergence, providing a systematic way to organize and interpret binary data features into meaningful clusters.

The clustering method does not make any assumptions about the number of feature clusters. After identifying the features associated with each cluster, each feature is mapped to its corresponding bin. Features that are not considered rare (i.e., present in at least 20% of the data) are not mapped to any bin and are used as they are. Only the rare features are mapped to their corresponding cluster, forming the Cluster Map.

Using the Cluster Map, the features within each cluster are represented as a Feature Bin Matrix (FBM). The training FBM consists of the features in the corresponding cluster, along with the feature values for all patients in the training dataset (without the class label). The unsupervised learning [21] is performed on the training FBM using the same BMD algorithm, iteratively for each cluster in the Cluster Map. The resulting values for each cluster are obtained. The trained model is then used to predict the cluster labels for the test FBM.

The unsupervised bins engineered from the FBMs are merged with the prevalent features (with the features excluded from the Cluster Map) to form a new engineered Feature Matrix (FM). This process is carried out separately for the training and test sets, resulting in the train FM and test FM, respectively. During the data level clustering, both datasets are handled separately without the class label to prevent data leaks.

**Fig. 2:**
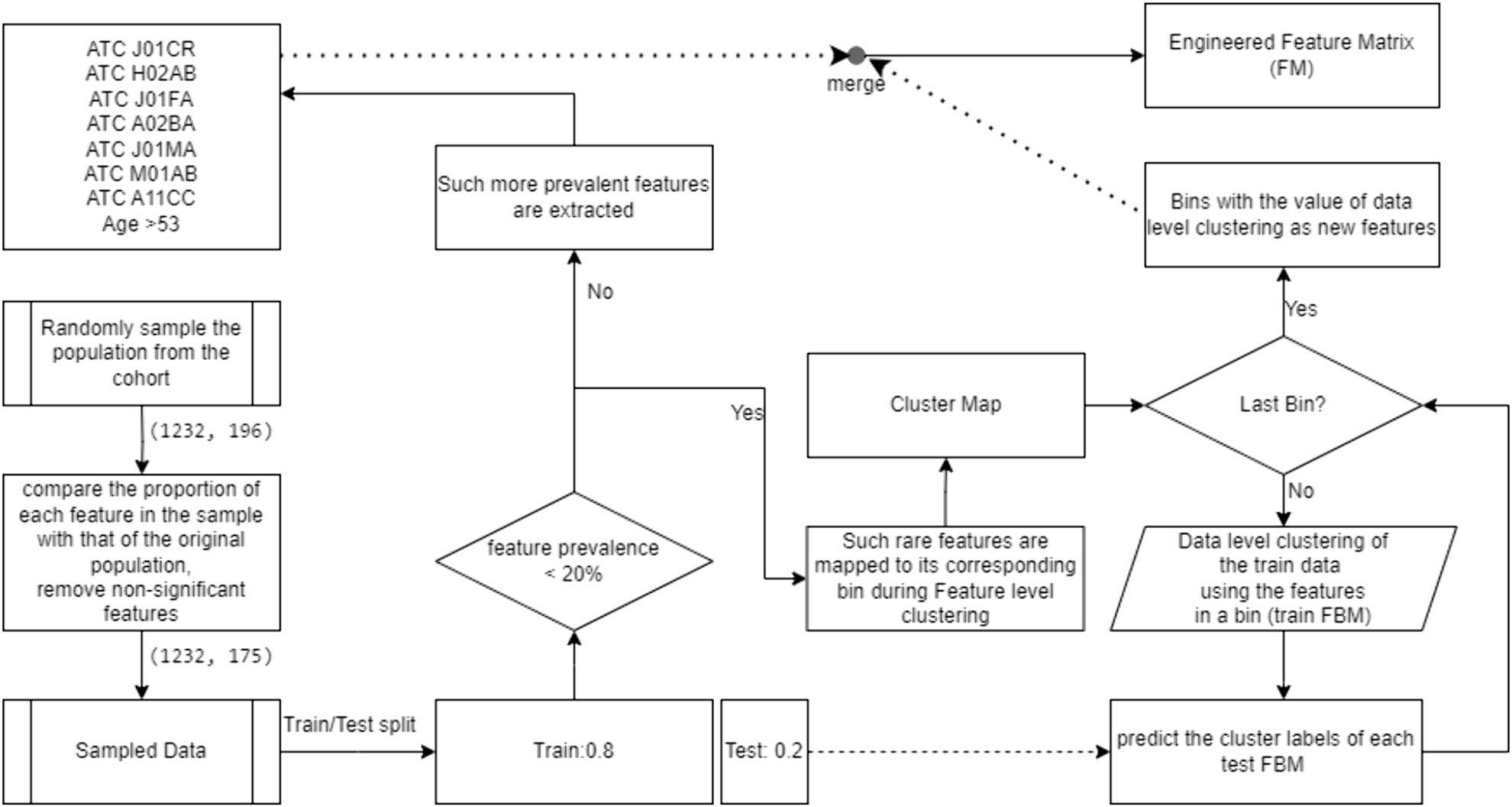
Unsupervised feature binning of rare features and generation of the Feature Matrix using new engineered features and other features: First of all data pertaining to prevalent features are sliced out. On the remaining data which contain the non-prevalent features, the clustering is applied. The process involves both feature-level clustering, where features are grouped into clusters using the BMD algorithm, and data-level clustering, where patients’ records are grouped into clusters. These tasks are interconnected as features within each cluster are used to create FBMs. Subsequently, data-level clustering is performed on these FBMs to assign patients’ records into clusters. Thus value obtained from data level clustering act as new features to replace original sparse data. The ultimate objective is to construct an engineered FM by combining these new bins with prevalent features, ensuring that both prevalent and combinations of non-prevalent features are considered for predictive modeling

In the data-level clustering step, we categorize patients’ records into two distinct clusters based on the features within each bin. Each patient’s record is assigned to one of the two clusters, ensuring a comprehensive grouping based on the relevant features within the given bin. For instance, consider a scenario where a cluster comprises n features in a bin. During data-level clustering, each patient’s record (row) in that bin is assigned a specific value. Consequently, this assigned value represents the contribution of that patient to the n features in that bin. After completing the data-level clustering for all patients, each bin accumulates values for every patient. Then that bin act as a new engineered feature where the data level clustering provided values for that feature. The entire procedure is illustrated in Fig. 2.

### Predictive modeling

To assess the performance of different Machine Learning algorithms in predicting hospital admission due to Covid-19, we utilized the train and test FM datasets. Since the data is labeled, we employed a supervised learning approach on this engineered dataset. The trained binary classification model was then applied to the holdout data to classify patients into one of two classes: whether or not hospital admission is required, taking into account their multimorbidity history.

Following the creation of the train and test FM datasets with the newly engineered features, we analyzed the variance of each feature. We trained the train FM using various Machine Learning algorithms available in the Pycaret package [22], employing 5-fold cross-validation.

Due to the sparsity of the data and the skewed distribution of value levels (0 or 1), certain levels may dominate others, resulting in insufficient variation to generate informative features. Therefore, during the Machine Learning-based predictive modeling, such non-informative features can be disregarded. The criteria for ignoring low-variance features [23] are as follows:

$$\begin{aligned} \frac{\text {number of unique values in a feature}}{\text {sample size}} < 10\% \end{aligned}$$

and

$$\begin{aligned} \frac{\text {number of most prevalent value}}{\text {number of second most prevalent value}} > 20 \end{aligned}$$

The best-performing model is selected by examining the mean area under the curve (AUC) score of each Machine Learning model. Later, the best model is evaluated using the test FM and the performance scores are reported.

**Table 1:** Cluster Map: Rare features are clustered and mapped to their corresponding cluster (Bins) after feature level clustering

**Bin 1**	**Bin 2**	**Bin 3**		**Bin 4**	**Bin 5**			**Bin 6**		**Bin 7**		**Bin 8**	**Bin 9**	**Bin 10**	**Bin 11**
**ATC**	**ATC**	**ATC**	**ICD**	**ATC**	**ATC**	**ICD**		**ATC**	**ICD**	**ATC**	**ICD**	**ATC**	**ATC**	**ATC**	**ATC**
A10BA	A07EC	A03FA	338	G03AA	C01BC	041	550	A07EA	038	B01AA	278	A02BX	A02AD	JO1DD	J01CA
A12AX	B02AA	A05AA	574	G03CA	C01BD	162	560	A10AB	174	B05BB	295	B03BB	A07AA		J01XX
B01AB	C03EA	A12AA	727	G03DB	C01DA	211	562	A10BB	218	C09BX	427	C03CA	B03AA		N06AB
B01AC	C09BA	A12BA	V58	J05AB	C02AC	241	571	C03AA	296	M05BA	455	C08CA	C07AB		R03BA
C09DA	J01AA	B03BA			S01EE	250	585	C03DA	301	N04AA	553	C09AA	H03AA		
M01AX	J01DC	C02CA		**ICD**		298	599	C07AG	454	N05AA	618	C09CA	J02AC		
R03AC	J01EE	C03BA		621		354	722	C07BB	473	N05AD	626	C10AA	M01AH		
R06AE	M04AA	C07AA				410	780	C10BA	518	N05AH	717	N02AA	N02AX		
	N06AA	C09BB				428	786	L01BA	592	S01EC	726	N02CC	N02BE		
	P01AB	C10AB				434	813	N01BB	735		812	N03AX			
	R03AL	C10AX				437	820	N02AJ	V54		996	N06AX			
		D05AX				438	V43	N03AF			998	R03AK			
		N02AB				440	V53	P01BA			V64	R06AX			
		N02BA				470	V56	R03BB							
		N03AE				482	V57	R03DA							
		N03AG				486		S01ED							

## Results

### Clustering

After applying feature-level clustering to the training data, a Cluster Map is generated. In this Cluster Map, rare features are clustered and assigned to their respective bins, resulting in 13 feature clusters. The bin values for each observation are calculated by determining the cluster label of the corresponding features in that bin. Table 1 illustrates the resulting 11 bins after excluding features with low variance.

Consequently, from an initial set of 174 input features, we have created a low-dimensional feature matrix consisting of 17 features. Even though mapping data with a higher dimension to a space of low dimension leads to some information loss [24], the predictive ability of the new data without sparsity can be an advantage for Machine Learning modeling.

### Model selection

To select the best model from various Machine Learning algorithms, we compared the AUC score of each Machine Learning model after executing a 5-fold cross-validation.

#### Using all features

During cross-validation using the train data with all 174 features, the best performance was obtained by LR (accuracy 0.72, AUC 0.76, F1-score 0.69), CatBoost Classifier (accuracy 0.72, AUC 0.76, F1-score 0.68), and Gradient Boosting Classifier (accuracy 0.72, AUC 0.76, F1-score 0.67).

#### Using the features which are reduced by clustering technique and ignoring the features with low variance

During cross-validation using the train data with only 17 features, the best performance was obtained by LR (accuracy 0.7, AUC 0.74, F1-score 0.68), LDA (accuracy 0.7, AUC 0.74, F1-score 0.66) and Ada Boost Classifier (accuracy 0.7, AUC 0.73, F1-score 0.67). The 5-fold cross-validation scores of each Machine Learning model are tabulated in Table 2.

**Table 2:** Score of the Machine Learning models obtained during 5-fold Cross Validation using reduced features

	Model	Acc^a^	AUC^b^	Recall	Prec.^c^	F1	TT^d^
**LR**	Logistic Regression	0.7015	0.7376	0.6186	0.7436	0.6752	2.410
**LDA**	Linear Discriminant Analysis	0.7025	0.7370	0.5781	0.7712	0.6605	0.008
**Ada Boost**	Ada Boost Classifier	0.6964	0.7347	0.6248	0.7315	0.6737	0.030
**NB**	Naive Bayes	0.6843	0.7305	0.5823	0.7345	0.6492	0.006
**RF**	Random Forest Classifier	0.6772	0.7301	0.6267	0.6980	0.6601	0.196
**CatBoost**	CatBoost Classifier	0.6853	0.7272	0.5800	0.7398	0.6490	0.674
**XGBoost**	Extreme Gradient Boosting	0.6761	0.7184	0.5900	0.7159	0.6451	0.402
**QDA**	Quadratic Discriminant Analysis	0.6772	0.7171	0.5701	0.7267	0.6387	0.008
**ET**	Extra Trees Classifier	0.6690	0.7155	0.6064	0.6947	0.6469	0.178
**GBC**	Gradient Boosting Classifier	0.6914	0.7147	0.5761	0.7507	0.6516	0.028
**LightGBM**	Light Gradient Boosting Machine	0.6843	0.7146	0.5962	0.7260	0.6541	0.258
**KNN**	K Neighbors Classifier	0.6569	0.7058	0.5537	0.7001	0.6162	0.422
**DT**	Decision Tree Classifier	0.6548	0.6522	0.5618	0.6956	0.6201	0.006
**Dummy**	Dummy Classifier	0.4975	0.5000	0.4000	0.1990	0.2658	0.006
**SVM**	SVM - Linear Kernel	0.5513	0.0000	0.9091	0.5393	0.6700	0.010
**Ridge**	Ridge Classifier	0.7025	0.0000	0.5781	0.7712	0.6605	0.006

^a^ *Acc* Accuracy Score obtained by the corresponding Machine Learning model

^b^ *AUC* Area under the ROC Curve

^c^ *Prec* Precision score

^d^ *TT * Time taken in seconds

### Model performance evaluation

After analyzing the cross-validation results, the top three models are selected based on their performance. To assess the predictive ability of these Machine Learning algorithms on the reduced data without sparsity, we applied them to predict the outcome of Covid-19 hospital admission using the test Feature Matrix (FM).

The performance metrics of the selected models on the test FM (holdout data) are as follows: LR (accuracy 0.72, AUC 0.77, F1-score 0.69), LDA (accuracy 0.7, AUC 0.77, F1-score 0.67) and Ada Boost (accuracy 0.7, AUC 0.77, F1-score 0.68). For a comprehensive overview, please refer to Table 3 for the complete set of results.

**Table 3:** Performance Evaluation of the selected Machine Learning models using Holdout data

Model	Acc	AUC	Recall	Prec.	F1
**LR**	0.72	0.77	0.63	0.76	0.69
**LDA**	0.70	0.77	0.59	0.76	0.67
**AdaBoost**	0.70	0.77	0.65	0.72	0.68

### Feature importance

Feature importance refers to the scores assigned to input features, which indicate their relative significance in making predictions. These scores provide insights into the importance of each feature in the data and the model. Feature importance helps not only in explaining the influential features but also in understanding the data and model better.

####  Feature importance score from the model coefficients

In linear algorithms such as LR and LDA, the predictions are calculated as a weighted sum of the observations, with the coefficients determined by the algorithm. In this context, negative coefficients indicate that as the value of a feature increases, the severity due to Covid-19 is predicted to decrease, suggesting no hospital admission. The features with negative coefficients in both LR and LDA algorithms are bin 2, bin 3, bin 4, bin 7, bin 10, J01CR, J01FA, and Age >53. On the other hand, features with positive coefficients have a positive association with the severity outcome. A higher negative coefficient indicates a stronger negative association between the input feature and the severity outcome. For example, if the value of a cluster or feature is 1, it suggests that most patients belonging to that cluster or feature category have a lower chance of severe Covid-19 outcomes, and vice versa. Conversely, in the case of a positive coefficient, if the cluster or feature value is 1, it indicates an increased likelihood of severe Covid-19 outcomes, and vice versa.

**Fig. 3:**
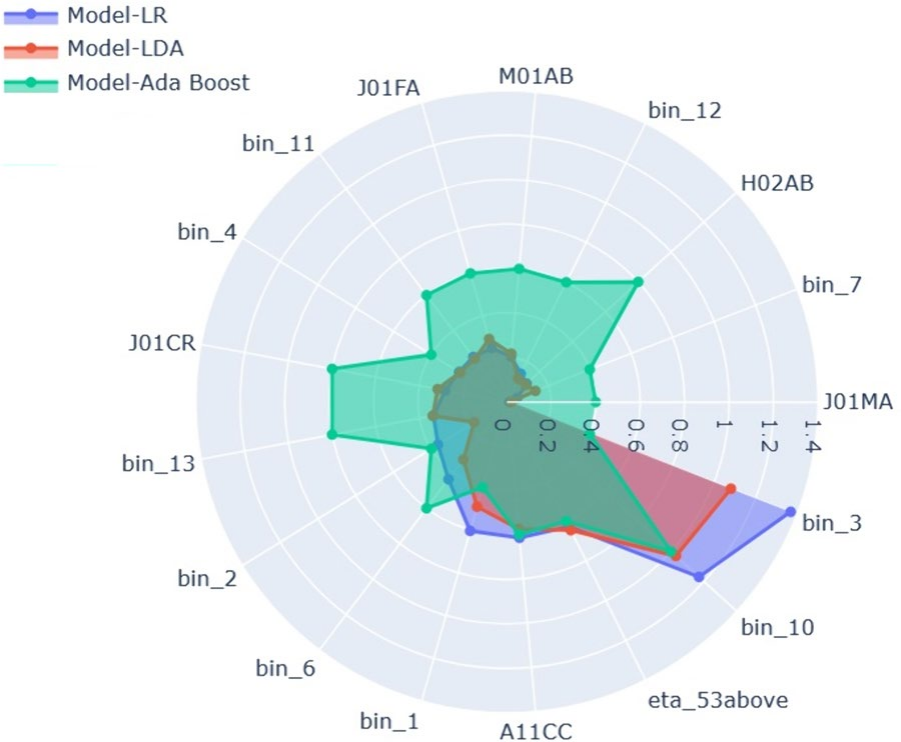
Feature importance scores from LR, LDA, and Ada Boost Models

#### The impurity-based feature importance

In the Ada Boost algorithm, the feature scores are determined using the Gini importance [25]. This score is calculated for each decision tree based on how much a single feature split improves the model’s performance, and it is normalized by the number of observations accounted for by that feature.

To analyze the feature importance of all three models (LR, LDA, and Ada Boost), we aggregated them and visualized the results in Fig. 3. In the case of linear models (LR and LDA), the feature importance is represented by the absolute values of the coefficients. For the Ada Boost Classifier, the feature importance values are scaled and presented in the visualization.

### Interpretation of the model

**Fig. 4:**
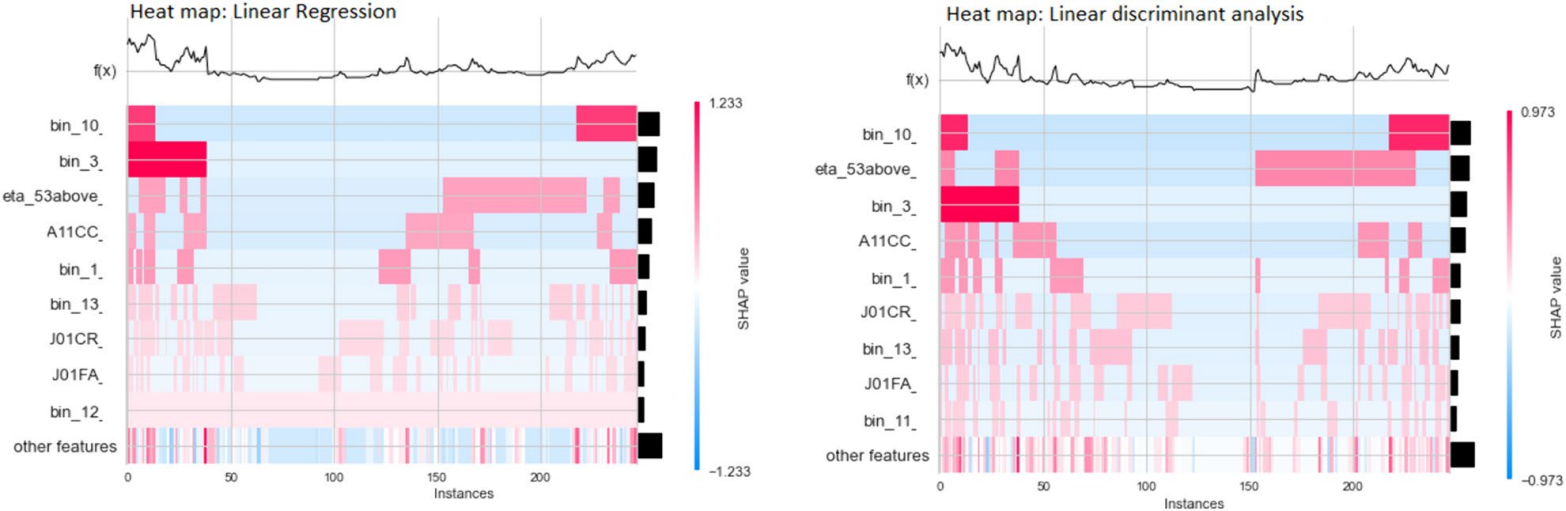
Heatmap matrix and global importance of features

We used SHAP (SHapley Additive exPlanations) [26] to interpret the most impactful features that our models utilize in determining the status of the hospitalization. The SHAP heatmap for the linear models depicted in Fig. 4 is based on the 20% test samples (X-axis). The sorted global feature importance is represented by the Y-axis and the bar plot (right-hand side). The magnitude of SHAP values of each observation (each patient) is represented by colors. The blue color for a feature denotes, in that patient profile, that particular feature has a value of 0 and this feature contributed to or impacted the prediction of the severity either positively or negatively. The topmost graph, f(x) represents the model predictions of each patient’s multimorbidity profile.

In the LR heatmap of SHAP values, while examining the f(x), the 0th patient observation number possesses a higher prediction. So, it is predicted that the patient is admitted to the hospital, and the features in cluster “bin 10” contribute more positively to the Covid-19 severity of that particular patient. Similarly, we can interpret the results of other patients for all the features using this visualization.

## Discussion

According to literature, women appear to be relatively less susceptible to SARS-CoV-2 infection than men [27]. But epidemiological data reveals no visible sex-based discrepancy in disease severity, suggesting that the progression of the virus is comparably favorable in both women and men, and there is a similarity in the age at which the rate of SARS-CoV-2 infection peaks for both male and female [28, 29]. However, the specific comorbidities that increase the risk of severe COVID-19 outcomes can differ significantly between men and women [30]. This underscores the need for a refined understanding of gender-specific factors influencing susceptibility and outcomes in the context of the COVID-19 pandemic. While existing literature provides valuable insights, there is a distinct lack of in-depth investigation specifically focusing on women [28]. To comprehensively address this gap in knowledge, it is imperative to advocate for targeted research works dedicated to understanding the unique aspects of women’s vulnerability or protection against COVID-19.

In our study, we utilized a combination of clustering and Machine Learning approaches to assess the severity of COVID-19 in women in midlife. Clustering less prevalent features into various Bins enhanced the interpretability of our data. By strategically grouping less common features into Bins and integrating them with prevalent ones, we aimed to capture a comprehensive picture of multimorbidity among women in midlife. Constructing clusters of multimorbidity and interpreting the outcomes at the patient level also allows us to identify, for future patients, which cluster value of a Bin contribute to whether a group of patients will be hospitalized or not due to COVID-19. Furthermore, in this study, identifying the most predictive feature or a Bin that includes less prevalent features helps in revealing the underlying combination of multimorbidity that predicts the severity of COVID-19 among women in midlife.

Examining Fig. 3 for the top-performing Machine Learning models in this study reveals that the age variable (age > 53) and the feature as a Anatomical Therapeutic Chemical (ATC) code, ATC A11C (Vitamin D and analogues) play crucial roles as predictors for COVID-19 hospitalization outcomes in middle-aged women. Existing literature provides support for the notion that individuals with a vitamin D deficiency are more susceptible to developing severe or critical cases of COVID-19 compared to those with sufficient vitamin D levels [31]. The other important features are the engineered features Bin 10 and Bin 3.

We found that Bin 10, which includes only one ATC code, ATC J01DD, consistently stands out as important across all major Machine Learning models. A retrospective drug-utilization study in Campania, southwestern Italy, found a higher prevalence of this third-generation cephalosporins (ATC J01DD) in COVID-19 positive adults under 40 and above 80, compared to the general population [32]. Additionally, a Hungarian study on hospital antibiotic consumption revealed a noteworthy 63.7% increase in the utilization of ATC J01DD from 2010 to 2019, with a substantial 70.46% surge during the pandemic years from 2019 to 2020 [33].

In Bin 3, under International Classification of Diseases, Ninth Revision (ICD-9), code ICD 574 corresponds to Cholelithiasis, a condition characterized by gallstone formation in the gallbladder. Research indicates a significant link between obesity and symptomatic gallstones, suggesting that even moderate overweight increases the risk in middle-aged women [34]. Individuals with gallstones often exhibit impaired gallbladder motility, potentially associated with additional gastrointestinal abnormalities [35]. Bin 3 also includes ATC A03FA (Propulsives) for stimulating gastrointestinal motility, ATC A05AA for medications related to bile acids and derivatives for managing certain liver diseases, ATC B03BA (Vitamin B12), ATC A12BA (Potassium), and ATC A12AA (Calcium). Calcium supplements for preventing mineral and bone disorders in chronic kidney disease (CKD) have been both praised and criticized [36].

Acute cholecystitis (AC) is a prevalent gastrointestinal ailment. The primary cause of AC is gallstone-related, but it may also be linked to diabetes, immunosuppression, CKD, and viral illnesses [37]. CKD significantly increases the risk of experiencing severe complications from COVID-19 [38]. A UK Biobank Community Cohort study revealed that in women both CKD and asthma posed a substantial risk for COVID-19 hospitalization, whereas in men, these conditions did not carry a similarly significant risk [30]. Additionally, females with asthma had a higher adjusted risk of hospitalization and death from COVID-19 compared to males with asthma, even after considering other factors [39]. Bin 3 also includes ATC D05AX (Corticosteroids in combination with vitamin D analogues for psoriasis treatment). Psoriasis may be associated with an increased risk of asthma, and childhood asthma is linked to a significantly higher risk of psoriasis [40].

Other features in Bin 3 include ICD 727 (Other disorders of synovium, tendon, and bursa) and ICD 338 (Pain, not elsewhere classified), encompassing chronic postoperative pain. A study on pain management during COVID-19 indicates that pain prevalence is 1.5-2 times more common in women than in men, with a higher ratio for specific conditions like fibromyalgia, which predominantly affects middle-aged women [41].

Research on the COVID-19 pandemic and cholecystitis suggests that the pandemic influenced healthcare-seeking behaviors for individuals with less severe health conditions [42]. A population-based cross-sectional study found that healthcare avoidance during the pandemic exhibited a robust correlation with being female, perceiving one’s health as fragile, and experiencing elevated levels of depression and anxiety [43].

Other medications in Bin 3 include Analgesics, Antiepileptics, and those related to the Cardiovascular system (C03, C07, C09, C10). In a study analyzing prescription data from June 2016 to March 2021, women exhibited a greater prevalence of antiseizure medication prescriptions compared to men, totaling around 1.3 million prescriptions [44]. Additionally, ATC C10AB, referring to fibrates, a type of medication used to lower cholesterol and triglycerides, may contribute to mitigating the inflammatory and thrombotic outcomes associated with SARSCoV-2 infection [45].

### Strengths and limitations

Training Machine Learning models in a reduced feature space would be beneficial, can be supported by our current results. Our intention in applying unsupervised methodology for feature reduction was rooted in the belief that a simplified feature space could lead to more interpretable models and potentially improved generalization performance. However, we recognize that the results obtained for Machine Learning models, which demonstrated superior performance with the complete set of features, appear slightly lower when trained on the reduced feature set.

Despite a slightly lower AUC, achieving competitive predictive performance with less number of features raises the question of the computational cost-effectiveness of using a reduced feature set. If computational resources are a critical consideration, our findings suggest that the reduced feature set could offer a pragmatic solution, providing a reasonable trade-off between predictive accuracy and computational requirements.

In the context of model interpretation, it is noteworthy to mention that, while writing this manuscript, SHAP support for AdaBoostClassifier is in the process of being integrated into the official SHAP library. A relevant pull request is under review on the SHAP GitHub repository [46]. We are closely monitoring the progress, and once integrated, we plan to incorporate SHAP plots for AdaBoost into our future analyses.

## Conclusion

The process of the unsupervised binning of the rare features can be divided into three phases: (1) extracting more prevalent features, (2) feature level clustering of the rare features to create bins, and (3) data level clustering of the features in a bin. The dimensionally reduced data with newly engineered features are used for the predictive modeling. The removal of data sparsity by this unsupervised binning of the rare features offered a low dimensional feature matrix for the predictive modeling. We have compared the predictive ability of the new sparsity-free feature matrix and the original sparse data and found that with a very low number of features itself, the model achieves nearly equal prediction performance. We have also checked the predictive utility of the new feature matrix by interpreting the feature importance and impact of the new features in the Machine Learning model.

The use of the method to address data sparsity in medical data and improve the understanding of the factors associated with the impact of infectious diseases on health outcomes in a population with multimorbidity is significant. By clustering sparse medical data and creating new features, the method could provide a more detailed understanding of multimorbidity patterns and the associations between different diseases. Improving the understanding of the factors associated with the severity of COVID-19 in this population could have important implications for public health policies, as middle-aged women with multimorbidity may be particularly vulnerable to the disease. The method has the potential to lead to better healthcare outcomes and inform public health policies related to COVID-19.

In our future works, we aim to enhance our work by integrating patient stratification based on their healthcare requirements, which entails the clustering of patient data to identify groups with similar healthcare utilization patterns. This approach will aid in identifying patient subgroups with distinct clinical profiles, which can help in designing targeted interventions and personalized care. Also, more research is needed to understand the direct impact of COVID-19 on midlife women’s help-seeking behaviors related to menopause specifically [47]. As a future work we are also planning to use Machine Learning to identify patterns in healthcare seeking behaviors before and after COVID-19 diagnosis in midlife women.

## Data Availability

The datasets are not publicly available. The requirement for informed consent is deemed unnecessary according to national regulation “Presidential Decree N. 20 of the Official Gazette of the Italian Republic N. 122 of May 26, 2022, “National Statistical Program” [48]. TLS\P (Turin\Piedmont Longitudinal Study) is a specific project of the Italian National Statistical Program (PSN) that is proposed by the National Statistical System (SISTAN), a network of public and private entities that provides the country and international bodies with official statistical information1, and is yearly approved by law by the Italian Parliament. In particular, since 2003 a specific form (PIE-00001 “Monitoring of socio-economic differences in mortality and morbidity through longitudinal studies”) is included in the PSN currently in effect for the three-year period 2020-2022 [49] and recently renewed for the period 2023-2025. Due to the restrictions imposed by ethical committees, the raw data cannot be made publicly or freely available to ensure the privacy and protection of individual-level data. However, researchers can request access to aggregated data by contacting the corresponding author through a reasonable inquiry.
